# Realization of optical logic gates using on-chip diffractive optical neural networks

**DOI:** 10.1038/s41598-022-19973-0

**Published:** 2022-09-21

**Authors:** Sanaz Zarei, Amin Khavasi

**Affiliations:** Department of Electrical Engineering, Sharif University of Technology, Tehran, Iran

**Keywords:** Engineering, Electrical and electronic engineering, Integrated optics, Metamaterials, Silicon photonics, Nanophotonics and plasmonics, Photonic devices

## Abstract

Optical computing is highly desired as a potential strategy for circumventing the performance limitations of semiconductor-based electronic devices and circuits. Optical logic gates are considered as fundamental building blocks for optical computation and they enable logic functions to be performed extremely quickly without the generation of heat and crosstalk. Here, we discuss the design of a multi-functional optical logic gate based on an on-chip diffractive optical neural network that can perform AND, NOT and OR logic operations at the wavelength of 1.55 µm. The wavelength-independent operation of the multi-functional logic gate at seven wavelengths (over a bandwidth of 60 nm) is also studied which paves the way for wavelength division multiplexed parallel computation. This simple, highly-integrable, low-loss, energy-efficient and broadband optical logic gate provides a path for the development of high-speed on-chip nanophotonic processors for future optical computing applications.

## Introduction

Modern semiconductor-based electronics is rapidly approaching fundamental limits caused by interconnect delays and large heat generation^1^. Because photons have intrinsically higher information-carrying capacity and produce low heat loads, photonic devices and circuits can potentially surmount these obstacles, but the goal of all-optical computing has not been achieved yet^1^. Optical logic gates are crucial building blocks for all-optical computing and they enable many applications like ultrahigh-speed information processing and all-optical networks. There are two major approaches toward all optical logic gates; one is based on the nonlinear optical effects^2–10^, especially the third-order nonlinear susceptibility, while another approach is based on the linear optical effects^11–19^ such as multi-beam interference^12–18^. However, the inherent instability of the interference-type optical logic circuits (including linear and nonlinear interference) hindered their application. Because they are heavily dependent on the precise control of the basic properties of the two input light signals, the control light and/or the pump light and precise controlling of the basic properties including phase difference, polarization, intensity and size of the input light beams (in the case that the two nanowires are close to each other, such as for the plasmonic logic gate) is difficult to implement^20^. Also, many of the reported works that are based on nonlinear optics, are severely limited by small nonlinear susceptibility of conventional materials which bounds their operation speed, consumption energy and device size. On the other hand, they have difficulty to be integrated with silicon-based optical devices. Furthermore, most of the reported works suffer from some certain fundamental limitations including big size, very few logic functions and even single function, having a big loss which makes them hard for integration, and more importantly narrow operating bandwidth. Therefore, it is desirable to achieve full logic functionality in a simple and compact photonic system which is stable, robust and suitable for monolithic integration, while at the same time is capable for wavelength multiplexed parallel computations (which helps to realize the full potential of optical computing).

Optical Neural Networks (ONNs) uses photons instead of electrons for computation, which enables surmounting the inherent limitations of electronics and improves the energy efficiency, processing speed and computational throughput. In ONNs, the neuron functionality and interconnectivity can be implemented with optical and photonic devices and the nature of light propagation. Here an on-chip diffractive optical neural network (DONN) is utilized to perform optical logic operations. In this configuration, the encoded light at the input layer is decoded through the hidden layers (1D-metasurfaces). The 1D-metasurfaces, named as metalines, are trained to scatter the encoded light into one of two small specified areas at the output layer, one of which represents logic state "1" and the other stands for "0". It is possible to train a single diffractive optical neural network to realize all seven basic logic operations. As a proof of principle, three logic operations (NOT, AND, and OR) are demonstrated in a single DONN at the wavelength of 1.55 µm. Furthermore, wavelength-independent operation by seven wavelengths (over 60 nm bandwidth in the silicon-on-insulator platform) is also demonstrated, which leads to wavelength division multiplexed parallel computation.

## Design and modeling

This section starts with the design principle of the multifunctional optical logic gate (“Design principle” section). Thereafter in “Architecture design” section, the optical architecture utilized for implementing the on-chip diffractive optical neural network is introduced. Subsequently, the theoretical model used to train the diffractive optical neural network on the computer is described in “Modeling” section. “Design considerations” section discusses the reasons of existing errors in the theoretical modeling of the system and possible solutions to mitigate the errors.

### Design principle

**Figure 1:**
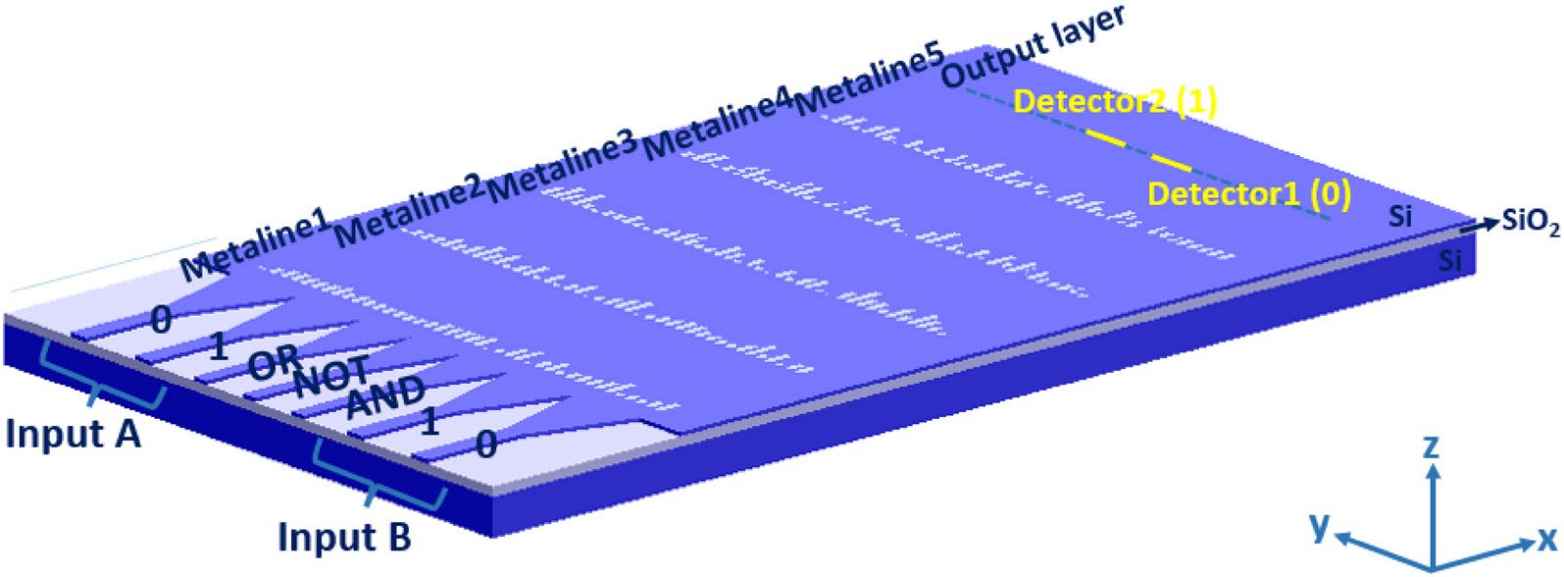
Schematic of on-chip diffractive optical neural network trained to perform optical logic operations AND, NOT and OR. Thera are seven input waveguide-tapers, three of which are for control signals AND, NOT and OR. Metalines (1D-metasurafces) are the diffractive neural network hidden layers. Meta-atoms on each metaline act as neurons. There are two detectors at the output layer corresponding to the computing results “0” and “1”. The propagation direction is along x-direction.

Figure 1 summarizes the principle of our optical logic operations. An optical deep learning framework in which the neural network is physically composed of multiple layers of diffractive 1D-metasurfaces (metalines) is utilized to optically perform basic logic operations through the interference of transmitted light. Analogous to artificial neural network, a diffractive neural network composed of one input layer, at least one hidden layer and one output layer. The input signals will be loaded onto the corresponding input waveguides and propagated through the tapers into the slab waveguide. The input layer is regarded as the plane at which the tapers meet the slab waveguide. The hidden layers (metalines) are designed to decode the encoded input light and form the image of the calculated output at the output layer^20^. Metalines are composed of arrays of subwavelength meta-atoms, which can be independently parameterized by an arbitrary number of variables ($w^{m}$). Each meta-atom behaves like an independent neuron in the neural network and interconnects to other meta-atoms of the adjacent layers through the diffraction of light^20^. After light exits the final metaline (the last hidden layer), it propagates a definite distance until it reaches the output layer of the network. The distance between two successive metalines and the distance between the last metaline and the output layer are also the design parameters. The output layer of the network consists of two detector regions (corresponding to the computing results "0" and "1") arranged in a linear configuration. For binary optical logic operations, the output gain only two values, "0" and "1", which can be considered as a classification task in machine learning^20^.

For our architecture, the physical structural parameters that accomplish the diffraction and prediction are designed in advanced. Initially, the parameters of the neural network are trained on the computer and then these parameters can be transferred to the physical structure.

As a proof of principle, we train the network such that it can perform NOT, AND and OR operations, but the network can be trained such that it is able to perform all seven logic operations. For such a network, seven input waveguides are required. Two waveguides for input A (one waveguide corresponding to "0" and one waveguide corresponding to "1"), two waveguides for input B, and three waveguides for control signals indicating NOT, AND, and OR (see Table 1). If it was desired that the network to be trained and perform as all seven logic operators, then seven waveguides should have been devised for control signals (one for each logic operation). For simplicity, it is assumed that when an input is applied to one of the waveguides, its value at the exit of the taper corresponding to that waveguide (or equivalently at the input layer) is "1".

**Table 1:** The optical I/O table for optical logic gate operations performed by diffractive optical neural network (DONN).

Input A		Input B		AND	NOT	OR	Detector1	Detector2
W0	W1	W0	W1					
1	0	1	0	1	0	0	1	0
1	0	0	1	1	0	0	1	0
0	1	1	0	1	0	0	1	0
0	1	0	1	1	0	0	0	1
1	0	0	0	0	1	0	0	1
0	1	0	0	0	1	0	1	0
1	0	1	0	0	0	1	1	0
1	0	0	1	0	0	1	0	1
0	1	1	0	0	0	1	0	1
0	1	0	1	0	0	1	0	1

### Architecture design

**Figure 2:**
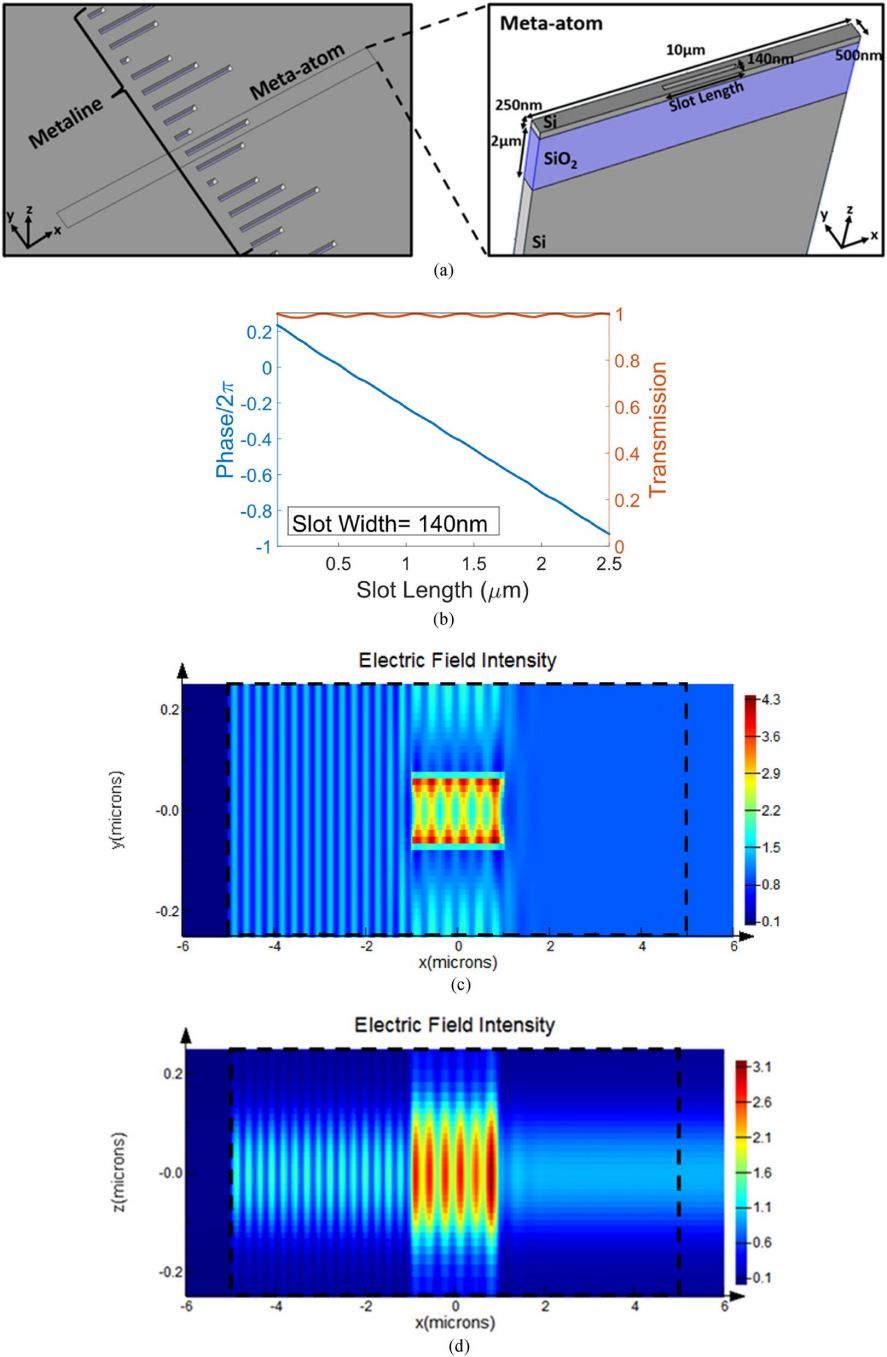
(**a**) Schematic of a metaline and a meta-atom consisting of a subwavelength slot which the length of the slot is a learnable parameter, (**b**) by changing the slot length between 100 nm and 2.3 µm, while keeping the slot width and height constantly as 140 nm and 250 nm, the transmission phase can be continuously tuned in the 0-to-2π range, while the transmission amplitude is higher than 0.96, (**c**) x–y view of the electric field intensity in a meta-atom with a 2 µm-length slot across the middle plane of the silicon slab (z = 0), (**d**) x–z view of the electric field intensity in a meta-atom with a 2 µm-length slot across the middle plane of the slot (y = 0).

The optical architecture used in this work is based on an SOI (silicon-on-insulator) platform which consists of several metalines; each metaline consists of a series of meta-atoms^21^. Each meta-atom in the metalines (hidden layers) is represented by a subwavelength slot (Fig. 2a) and the geometrical parameters of the slot are design parameters (some of them can be set as learnable parameters in training process). Therefore, each slot can be regarded as a weight element that connects to its adjacent layers through diffraction and interference of the in-plane waves. By adjusting the width and length (and height) of each slot, the fully control over transmitted amplitude and phase delay of a meta-atom can be achieved.

A silicon-on-insulator platform with a 250 nm silicon top layer and a 2 µm buried oxide layer is chosen. The lattice constant of the metalines is fixed to 500 nm (approximately one third of the wavelength) and the width and height of slots are constantly chosen as 140 nm and 250 nm, respectively. By changing the slot length between 100 nm and 2.3 µm, the transmission phase can be continuously tuned from 0-to-2π, while the transmission amplitude is higher than 0.96 (Fig. 2b). The design wavelength is 1.55 µm. The results presented in Fig. 2b are achieved using the commercial software package Lumerical FDTD, while FDTD ports with a fixed distance of 10 µm along x-direction are utilized to calculate the S-parameters of a meta-atom. The injection axis of the ports is x-axis and the fundamental TE mode is selected for excitation. The x–y view of the electric field intensity profile across the middle plane of the silicon slab (z = 0) and x–z view of the electric field intensity profile across the middle plane of the slot (y = 0) for TE wave tunneling through a 2 µm-length slot in a meta-atom are illustrated in Fig. 2c,d, respectively.

During the training process, the lengths of slots are considered as learnable parameters and the training can proceed based on both transmission phase and amplitude of meta-atoms. However, for simplifying the design process in this article, the simultaneous adjustment of amplitude and phase of meta-atoms is avoided and only phase modulation is taken into account with transmission efficiency being set to 1, which doesn’t appreciably affect the accuracy of the resulting design due to near-1 transmission amplitude of meta-atoms.

### Modeling

#### Forward propagation

The multiscale nature of metasurfaces, which the whole metasurface has macroscale dimensions and its subwavelength scattering elements have nanoscale length scales, make using full-wave simulation tools to be computationally expensive or even prohibitive due to memory requirements. Locally periodic approximation that assumes the metasurface is locally periodic (i.e., is periodic over any small region) can be a high-speed alternative that calculate the field across the plane right after the metasurface, by using small full-wave simulations to compute the field transmission phase and amplitude for each scattering element (meta-atom). Thereafter, by using near-to-far field transformation^22,23^, or scalar-wave approximation^24,25^, or spatial domain electromagnetic propagation^26^, the fields can be propagated between metasurface layers. In this article, near-to-far field transformation^22,23^ is utilized to propagate the fields.

As our optical neural network is physically composed of multiple layers of diffractive 1D-metasurfaces (metalines) in the SOI platform, it can be assumed as a two-dimensional problem.

The wave-number that light travels in the silicon slab waveguide is $k = n_{eff} .\frac{2\pi }{\lambda }$, where $n_{eff}$ is the effective refractive index of the silicon slab waveguide associated with the fundamental TE mode. It is assumed that each metaline is composed of N meta-atoms and the input electric field ($E^{{in,\lambda_{j} }}$) to the meta-system is pixelated to N pixels. Thus, $E^{{in,\lambda_{j} }}$ is an N × 1-dimensional vector. Each meta-atom on a single metaline layer introduces a phase and amplitude modulation on the input electric field at that meta-atom. By using full-wave simulations to obtain the phase and amplitude modulation of each meta-atom (as is done in “Architecture design” section), the electric field along the line right after each metaline can be achieved as:

1 $$E_{m}^{{out,\lambda_{j} }} = T_{m}^{{\lambda_{j} }} \exp (i\phi_{m}^{{\lambda_{j} }} )E_{m}^{{in,\lambda_{j} }}$$

where $E_{m}^{{in,\lambda_{j} }}$ is the input electric field to the *m*'th metaline, and $E_{m}^{{out,\lambda_{j} }}$ is the electric field along the line right after the *m*'th metaline, and $T_{m}^{{\lambda_{j} }}$ and $\phi_{m}^{{\lambda_{j} }}$ are diagonal matrices containing the transmission amplitudes and phases of meta-atoms on layer m at the wavelength $\lambda_{j}$. Now that the near-field output of each layer ($E_{m}^{{out,\lambda_{j} }}$) is known, the far-field output can be calculated through a near-to-far field transformation:

2 $$E_{m + 1}^{{in,\lambda_{j} }} (\vec{y}) = - \int_{line} {2G(\vec{y},\vec{y^{\prime}})E_{m}^{{out,\lambda_{j} }} } (\vec{y^{\prime}})d\vec{y^{\prime}}$$

where $E_{m + 1}^{{in,\lambda_{j} }}$ is the far-field output of *m*'th metaline that is the input electric field of (*m* + *1*)'th metaline, $G(\vec{y},\vec{y^{\prime}}) = - \frac{ik}{4}H^{(1)} (kr)\hat{n} \cdot \frac{{\vec{r}}}{r}$ is the Maxwell Green's function (where $H^{(1)}$ is the Hankel function of the first kind, $k = n_{eff} .\frac{2\pi }{\lambda }$, $\vec{r} = \vec{y} - \vec{y^{\prime}}$, $r = \left| {\vec{r}} \right|$ and $\hat{n} = \hat{x}$ is the surface unit-normal vector). This integral can be changed to summation and then converted to matrix multiplication as:

3 $$E_{m + 1}^{{in,\lambda_{j} }} = GE_{m}^{{out,\lambda_{j} }}$$

where $G_{ij} = G(\vec{y}_{i} ,\vec{y^{\prime}}_{j} )$, $E_{m}^{{out,\lambda_{j} }} (j) = E_{m}^{{out,\lambda_{j} }} (\vec{y^{\prime}}_{j} )$ and $E_{m + 1}^{{in,\lambda_{j} }} (i) = E_{m + 1}^{{in,\lambda_{j} }} (\vec{y}_{i} )$. Multiple Fresnel-like reflections of the intermediate metaline layers are neglected due to the near-1 transmission amplitude of our meta-atoms. At the output layer of the network, the output electric field ($E_{{}}^{{out,\lambda_{j} }}$) can be calculated following a series of matrix–vector multiplications as described above. $E_{{}}^{{out,\lambda_{j} }}$ is the function of the learnable parameters $\left\{ {w^{1} ,w^{2} ,...,w^{M} } \right\}$ of the M metalines. Once $E_{{}}^{{out,\lambda_{j} }}$ is computed, the output intensity can be calculated as $I^{{out,\lambda_{j} }} = E^{{out,\lambda_{j} *}} E^{{out,\lambda_{j} }}$.

#### Backpropagation

For backpropagation, a cost function is specified in terms of the squared errors between a desired set of output intensity distributions and realized set of intensity distributions at a given iteration:

4 $$C = \sum\limits_{j = 1}^{J} {\sum\limits_{k = 1}^{K} {\sum\limits_{s = 1}^{N} {\left( {I_{s}^{{\lambda_{j} ,f_{k} }} - I_{s}^{{des,\lambda_{j} ,f_{k} }} } \right)^{2} } } }$$

where the squared error is summed for J input wavelengths, K input field distributions at a given wavelength, and N sample points along the output line. The cost function is iteratively minimized by adjusting the learnable parameters $\left\{ {w^{1} ,w^{2} ,...,w^{M} } \right\}$. For back-propagating the errors in the network to update these learnable parameters, the adjoint method, in which the gradient of the cost function with respect to all learnable parameters can be computed using only two full-field simulations^24^, is utilized, the details of which is given in supplementary note 1.

### Design considerations

One important issue in an optical neural network design is its final experimental inference capability. Most of the diffractive optical neural networks proposed up to now, show high percentage of consistency between numerical predictions and experimental verifications^26–30^. These DONNs are mainly designed for classification^26–28,30^. In the tasks of classification and regression, some amount of error is tolerable. However, logic gates should be highly immune from errors. So, for our design, the matching between numerical testing results and experimental testing results should be 100% for accurate and precise performance of multi-functional logic gate.

In^26^, two main problems which result in the difference between numerical predictions and full-wave electromagnetic simulations, are recognized. One problem is that the effective refractive index of an identical slot at different positions along the metaline is different, because the light inputs the slot at different locations with different angles. The other problem is the existence of mutual interference between adjacent slots of different lengths when the light inputs them at the same angle. The solution to these problems are also suggested in^26^. For the first problem, if the incident light enters the slots at a smaller angle, then a more stable effective refractive index can be achieved for all the slots along the metaline. For the second problem, in order to reduce the influence of mutual interference between adjacent slots, a slot group composed of multiple identical slots is used to approximate a neuron value.

**Figure 3:**
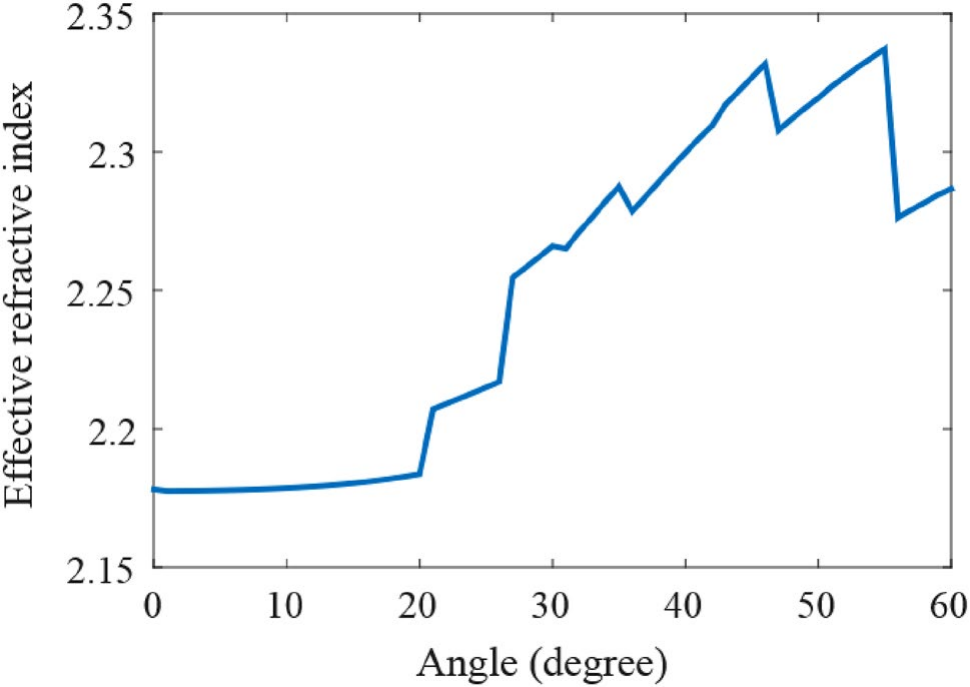
Effective refractive index of a slot with 2 µm length as a function of incident light angle.

For our architecture with lattice constant of 500 nm, width and height of the slots as 140 nm and 250 nm and an arbitrary-chosen value of 2 µm for slot length (within the range of 100 nm–2.3 µm), we calculate $n_{eff - slot}$ (effective refractive index of the slot) for a slot, when the incident light enters the slot from diverse angles (see Fig. 3). The effective refractive index of the slot can be calculated from^26^:

5 $$n_{eff - slot} = \frac{{\Delta \phi_{i} }}{{L_{slot} \cdot k_{0} }} + n_{eff - slab}$$

where $L_{slot}$ is the length of the slot, $n_{eff - slab}$ is the effective refractive index of the slab waveguide, $k_{0}$ is the wavenumber of light in the slots and $\Delta \phi_{i}$ is the phase delay generated by the slot. As is seen in Fig. 3, for all the incident angles of light to the slot which are less than 20˚, the effective refractive index of the slot remains nearly constant. Therefore, the distance between the metalines should be high enough, such that the incident light angle to the meta-atoms is less than 20˚.

**Figure 4:**
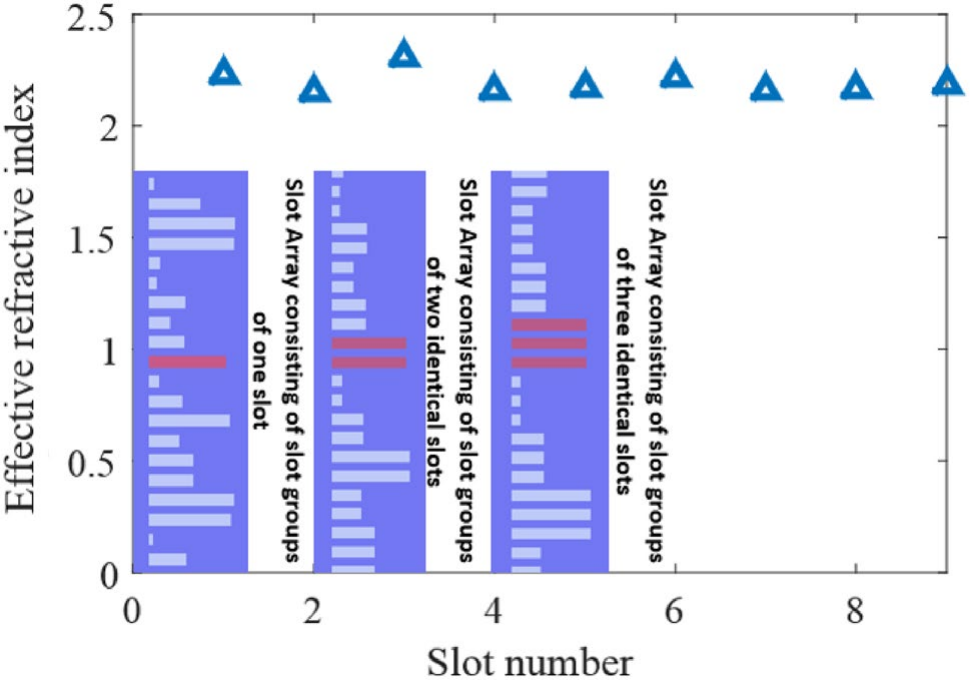
Effective refractive index of a slot group with different number of identical slots. For generating this figure, a slot array with 20 randomly-generated slot lengths is used. The effective refractive index is calculated for slot groups associated with the slot colored in red with the slot length of 1.964 µm.

Also for our architecture, the $n_{eff - slot}$ calculated by the phase delay generated by a slot group with a different number of slots is shown in Fig. 4. It can be seen that when the number of slots increases in a slot-group, the effective refractive index of the slot calculated by the phase delay generated by the slot groups tends to be more stable. Also, it can be inferred from Fig. 4 that when the slot group includes two identical slots, the calculated effective refractive index is very close to the final stable value. It should be mentioned that for generating Fig. 4, a slot array with 20 randomly-generated slot lengths is chosen. The effective refractive index is calculated for a slot with 1.964 µm slot length (marked in red in Fig. 4).

## Results

In this part, on-chip optical Boolean logic operations (AND, OR, and NOT) are first numerically demonstrated at the wavelength of 1.55 µm and thereafter the achieved results are verified using 2D FDTD and 2.5D FDTD variational solver of Lumerical Mode Solution. The meta-system consists of five metalines (hidden layers), with each metaline containing 100 meta-atoms (neurons). While the length of each metaline is 100 µm, the distance between two successive layers is 300 µm, and after light exits the fifth metaline, it propagates 300 µm until it reaches the output line of the network with two linearly-arranged detection regions which are representative of logic states "0" and "1". The length of each detection region is 4 µm and the center-to-center distance between the two regions is 12 µm. Also, it is assumed that the distance between the input layer and the first metaline is zero, while the thickness of each metaline is 10 µm.

As was stated in “Design considerations” section, for a design to perform accurately as a multi-functional logic gate, it is necessary that the full-wave simulation results provide 100% fit to the numerical testing results. Therefore, according to Fig. 3, the distance between the metalines should be such that the incident light angle to the meta-atoms along the metaline to be less than 20°, which means that

6 $$Distance\;\;between\;\;layers\;\; \ge \left( {\frac{100\;\upmu \text{m}}{{\tan (20^\circ )}} = 274\;\upmu \text{m}} \right)$$

Therefore, for this design, it is assumed that the distance between metalines as well as the distance between the fifth metaline and the output line is 300 µm. Also according to Fig. 4, each neuron value in this design is approximated as a slot group composed of two identical slots, in order to reduce the influence of mutual interference between adjacent slots.

**Figure 5:**
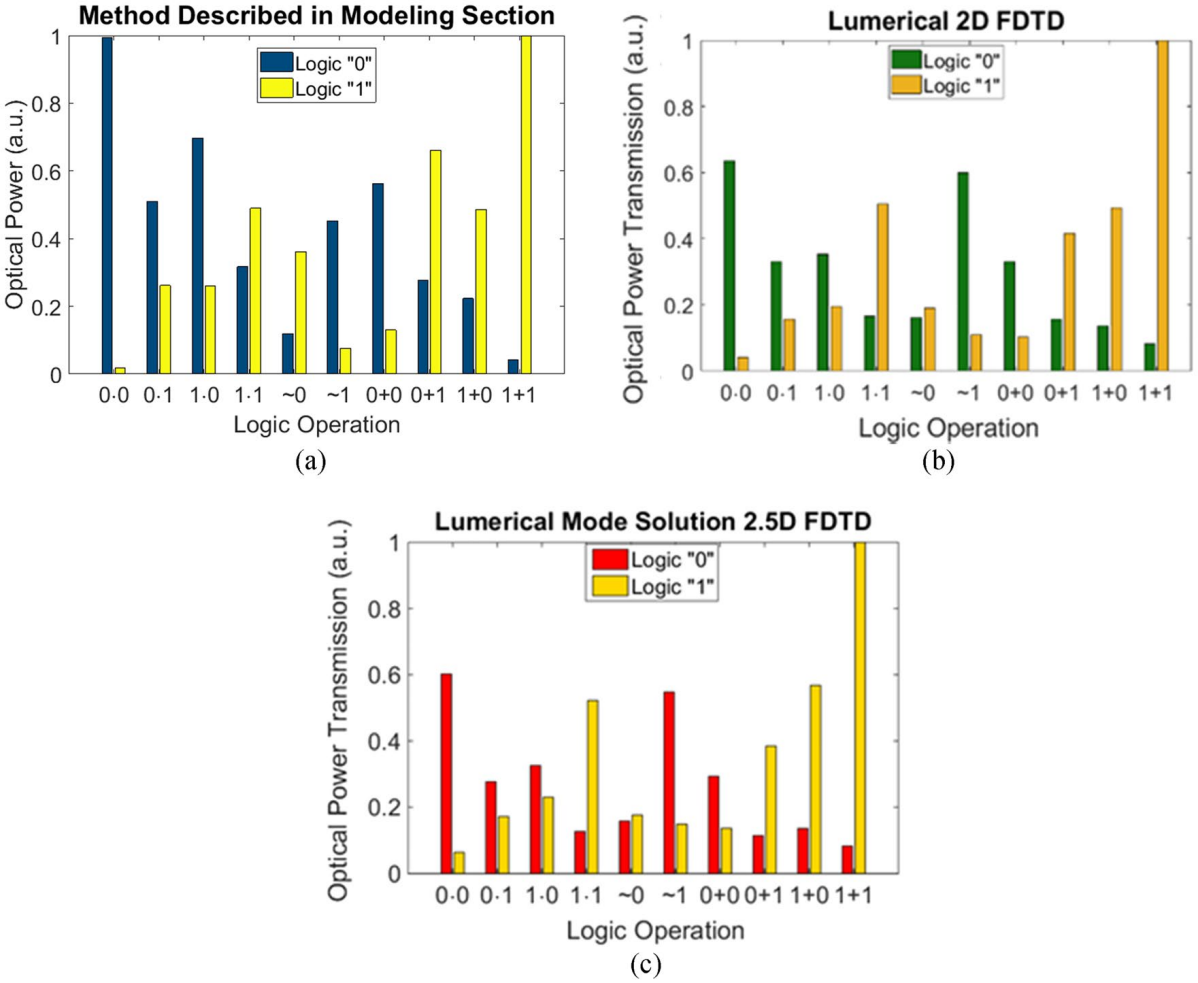
The logic operation of the numerically-trained diffractive model with five diffractive layers, (**a**) numerical results, (**b**) Lumerical 2D FDTD results, and (**c**) Lumerical Mode Solution 2.5D FDTD simulation results.

Training is performed using 10 input combinations (4 input field distributions for AND, 4 input field distributions for OR, and 2 input field distributions for NOT) and for each input combination, the desired intensity distribution $I^{{des,\lambda_{j} ,f_{k} }}$ is defined as a rectangle centered over appropriate output detection region with $\sum\limits_{s\;over\quad \det ection\quad region}^{{}} {I_{s}^{{des,\lambda j,f_{k} }} } = 1$ at the output layer of DONN. The optimization problem involves 500 design variables (100 variables per metaline). The lengths of slots (variables) are initially set to 1.1 µm in the optimization and due to fabrication constraints, the minimum slot length is specified as 100 nm and the maximum slot length is set as 2.3 µm in order that the transmission phase changes within 0-to-2π range (with minimum feature size of 100 nm, the fabrication of metalines is feasible utilizing current deep UV photolithography technique used in silicon photonics foundry). For a specific input, when the output signal is accurately distributed such that the total intensity upon the expected detector corresponding to that input has significant value comparing to the other detector, the classification can be considered successful. Using the mathematical framework described in “Modeling” section, the lengths of slots (and correspondingly the transmission phase of meta-atoms) are adjusted in search for minimum of the cost function which is defined as the squared errors between the desired set of output intensity distributions and realized set of intensity distributions at a training iteration. Figure 5a describes the numerical performance of the designed all optical logic gate. These results are verified with Lumerical 2D FDTD (Fig. 5b) and 2.5D FDTD variational solver of Lumerical Mode Solution (Fig. 5c). It should be mentioned that both numerical simulations and 2D FDTD simulations are two-dimensional and the effective refractive index of the silicon slab waveguide is used as the refractive index of background material in the simulations. However, 2.5D FDTD simulations consider the 3D nature of the diffractive optical neural network (DONN) structure (Lumerical 2.5D varFDTD method offers comparable accuracy and versatility to that of 3D FDTD, while only requiring the simulation time and memory of a 2D FDTD simulation^31^). As is seen in Fig. 5, 2D FDTD results and 2.5D FDTD results are very near to each other, although not completely identical. Therefore, it can be stated that 2D modeling of the structure considering effective refractive index of the silicon slab waveguide makes few discrepancies in the full-wave electromagnetic results. However, there is a significant difference between the predicted results by numerical simulations and full-wave electromagnetic simulations. The discrepancy between the numerical results (Fig. 5a) and Full-wave electromagnetic simulations (Fig. 5b,c) is dominantly due to the local periodic approximation that is used in the numerical modeling. Locally periodic approximation is alternatively utilized in many numerical modeling of metasurface-optics^22–26,32,33^, because of large memory requirements of full-wave simulators. Also, the assumption of unit-transmission-amplitude for all meta-atom geometries along metalines can cause some small discrepancies between the two results (the reason that we mention “small discrepancies” is that the transmission amplitude of all meta-atoms is very near to 1). Furthermore, multiple Fresnel-like reflections of the intermediate layers of the optical system is neglected in our numerical simulations, however, due to near-to-1 transmission amplitude of meta-atoms, the reflection from intermediate metalines is not significant.

Because of computational restrictions of full-wave simulators such as Lumerical, here we choose a very compact structure with as few neurons as possible to make the structure simulable with such a commercial package for the purpose of verification. In this case, the contrast ratios between the measured intensities of two designated regions drops. Higher contrast ratios can be achieved utilizing higher number of neurons in each metaline.

It is noteworthy to mention that it is possible to train the diffractive optical neural network to perform any other logic operations like NAND, NOR, XOR and XNOR or even all the seven basic logic operations at the same time. Depending on the number and complexity of the tasks that the neural network is trained for, the number of neurons, the number of diffractive layers, and the distance between layers may subject to change. Furthermore, it is possible to design the multifunctional logic gate such that it can perform other functions as well which ease the integration of the gates with other devices.

**Figure 6:**
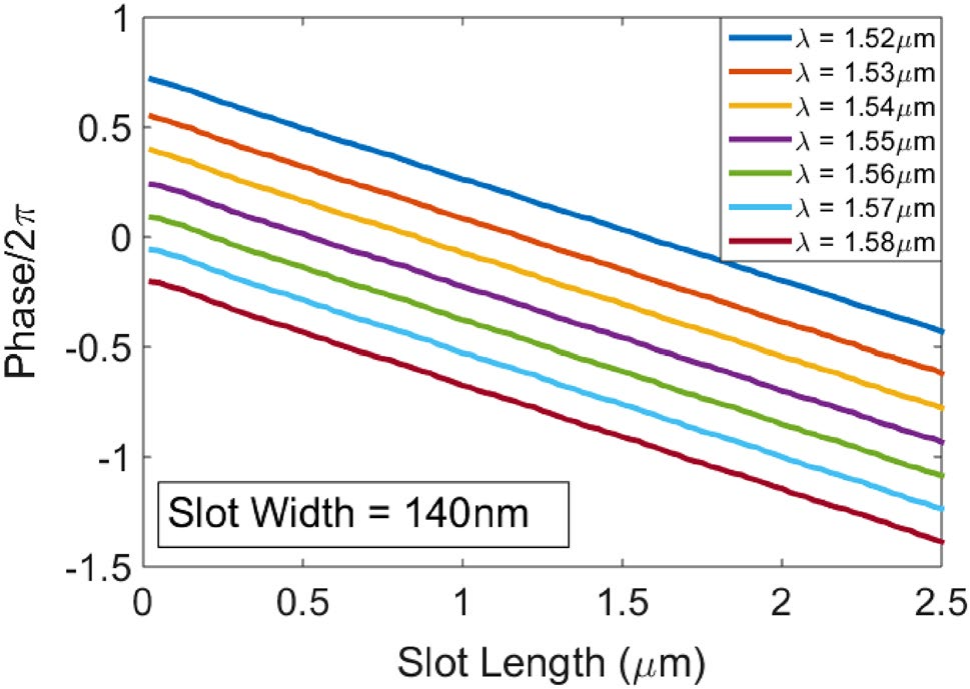
The wavelength-dependent phase shift of a meta-atom versus slot-length, fixing the slot width and height at 140 nm and 250 nm respectively, calculated by Lumerical FDTD.

Also, we have demonstrated wavelength division multiplexed parallel computation at seven different wavelengths (1520, 1530, 1540, 1550, 1560, 1570, and 1580 nm). Figure 6 shows the λ-dependent transmission phase response of meta-atoms versus slot length when the slot width is fixed at 140 nm. Lumerical FDTD is exploited to calculate the λ-dependent transmission phase of meta-atoms. The distance between FDTD ports is again set to be 10 µm along the x-direction. Again, training is performed using 10 input combinations at seven different wavelengths in the 1520–1580 nm range. In this case, the cost function should be computed for 7 input wavelengths and 10 input field distributions at a given wavelength and 100 sample points along the output line according to Eq. (4).

**Figure 7:**
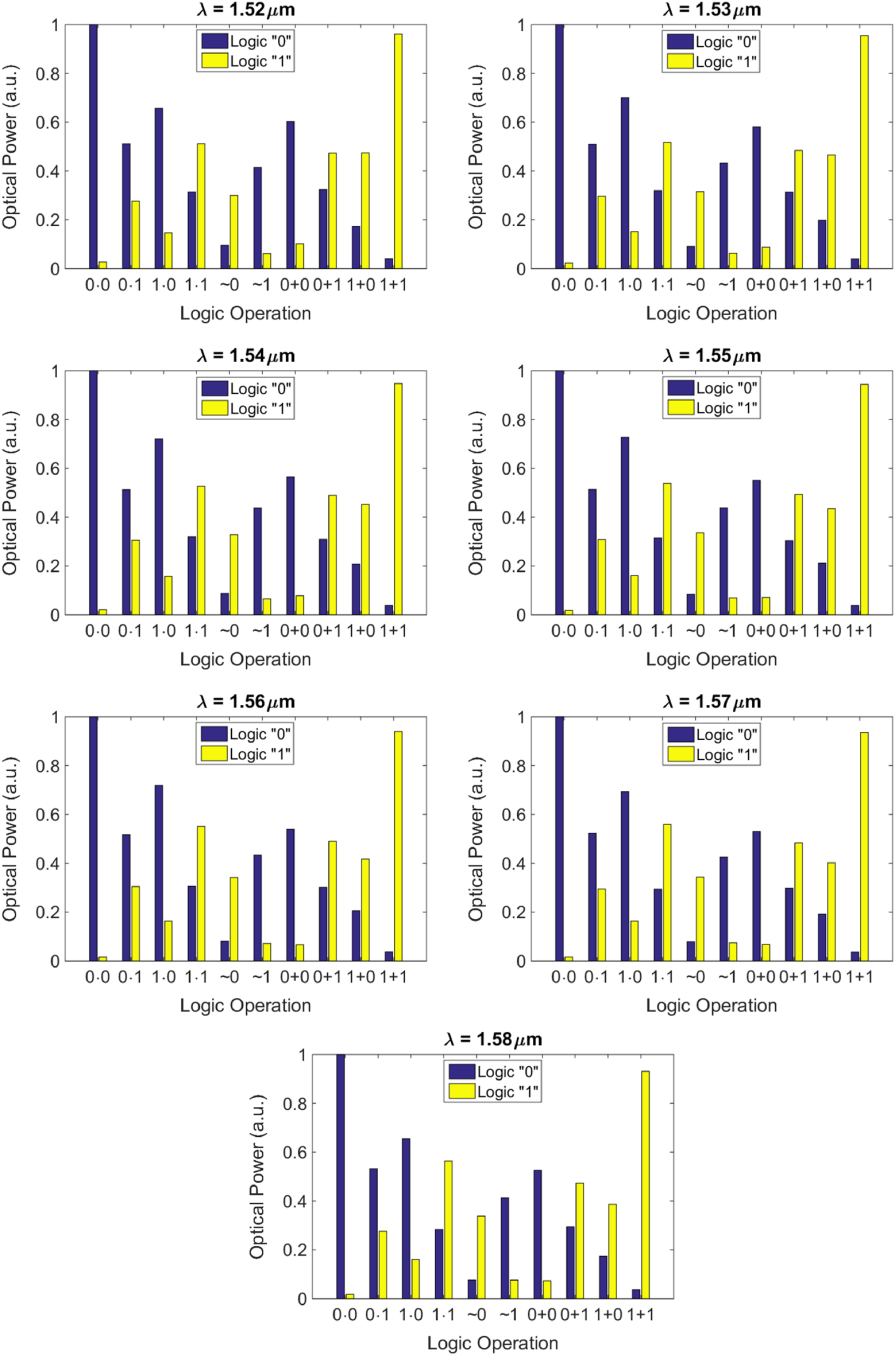
The logic operation of the numerically-trained diffractive model with five diffractive layers at seven different wavelengths in the 1520-1580 µm range. The design parameters of this diffractive model is similar to the design depicted in Fig. 5.

Principally in training, wavelength-dependent phase response of the meta-atoms (Fig. 6) and therefore wavelength-dependent phase response of the metalines and the wavelength-dependent refractive index of silicon and thus the wavelength-dependent effective refractive index of the guided light confined in the silicon slab should be taken into account. $I^{{des,\lambda_{j} ,f_{k} }}$ is defined as before for each wavelength. A separate forward and backward propagation is performed for each of the seven wavelengths. The gradients computed for each wavelength are summed to obtain the overall gradient of the cost function with respect to all of the design variables. All other design parameters are like the ones in the training of the single-wavelength DONN of Fig. 5. The numerical performance of the wavelength-independent DONN at 1520–1580 nm range as an all-optical multi-functional logic gate is depicted in Fig. 7. This provides the capability to perform simultaneously different operations for each wavelength channel by varying the input conditions which means that totally independent parallel logic operations is possible up to the number of input channels with a single gate^12^. Due to huge computational time required to verify all these results, they haven't been verified with 2.5D FDTD variational solver of Lumerical Mode Solution.

## Discussion

### Design verification

Because of computational restrictions encountered in a single simulation of our metasystem using full-wave simulation tools as was described in “Modeling” section, it was tried to choose an optical gate design that is as compact as possible. However, the challenge was designing an optical logic gate with 100% consistency between its numerical performance and the performance it shows when simulated with a commercial full-wave electromagnetic software. On the path we took to achieve full compliance between the two simulation results, we numerically trained a number of diffractive networks and verified their performance with Lumerical 2.5D FDTD. Table 2 reports the results of these investigations.

**Table 2:** The characteristics of a number of diffractive neural networks that operate as a multi-functional logic gate and the matching between their numerical testing results and full-wave simulation results.

Design	Number of layers	Number of neurons in each layer	Number of slots in each neuron	Length of the layers (µm)	Distance between layers (µm)	Distance between the last layer and output layer (µm)	Length of detection regions (µm)	Center to center distance between detection regions (µm)	Percentage of matching between numerical predictions and full-wave simulations (%)
1	3	200	1	100	200	200	4	12	90
2	3	100	2	100	200	200	4	12	70
3	3	200	1	100	300	300	4	12	90
4	3	100	2	100	300	300	4	12	80
5	5	200	1	100	50	50	4	12	80
6	5	200	1	100	200	200	4	12	90
7	5	100	2	100	200	200	4	12	70
8	5	200	1	100	300	300	4	12	100
9	5	100	2	100	300	300	4	12	100
10	3	200	1	100	200	200	4	32	80
11	3	200	1	100	200	200	4	22	90
12	5	200	1	100	50	50	4	32	60
13	5	200	1	100	200	200	4	22	90
14	5	100	2	100	200	200	4	22	80

It can be inferred from the results reported in Table 2 that approximating a neuron value by a slot group composed of two identical slots doesn’t necessarily lead to better matching between the numerical results and full-wave simulation results. The main reason is that the number of meta-atoms in the metasystem composed of slot groups is twice less than the metasystem composed of individual slots. Also, according to Fig. 4, in our chosen architecture, using a slot group of two identical slots doesn’t make a significant difference in the effective refractive index of the slot comparing to when using an individual slot. So, this was not unexpected.

This issue is evident when comparing designs 1 and 2, designs 3 and 4, designs 6 and 7, as well as designs 13 and 14, which only differ in the number of slots in a meta-atom (and therefore the number of meta-atoms in each metaline). In addition, despite the fact that both designs 8 and 9 show 100% consistency between numerical testing results and 2.5D FDTD results, design 8 shows better consistency especially in terms of contrast ratios between logical values “0” and “1” comparing to design 9. Also, by comparing designs 2 and 4, designs 6 and 8, and designs 7 and 9 in Table 2, it can be deduced that increasing the distance between the layers from 200 to 300 µm results in better fitting of the numerical and full-wave simulation results. For a more detailed discussion on the design verification, please refer to supplementary note 2 and supplementary Table S1.

### Design performance

Falling under the category of diffractive neural networks, the presented design has most of the advantages that multi-functional optical logic gate design proposed in^20^ based on diffractive neural network actually has, that are mainly the non-requirement to precise control of the input light beams which reduces the instability of optical logic circuits and the capability of complete logic functionality in a single optical network (all seven logic operations) which makes the logic gates easy for device fabrication and system integration (see Table 3 for more details).

**Table 3:** Comparison between our design and the multifunctional optical logic gate based on DONN presented in^20^.

Ref.	Device architecture	Gate	Operation wavelength	Dimensions	Bit rate	Number of neurons	Contrast ratio (dB)	Operation bandwidth
^20^	Diffractive optical neural network	AND, OR, NOT (with the capability to be trained for all seven logic operations)	17.6 mm	301 mm × 421 mm × 1420 mm	–	2520	> 9.6	–
This work	On-chip diffractive optical neural network	AND, OR, NOT (with the capability to be trained for all seven logic operations)	1.55 µm	100 µm × 1850 µm^a^	20 Gbps^b^	500	< 10.82 (Based on 2.5D FDTD simulation results)	60 nm (capable to be trained for larger bandwidths)

^a^For a brief discussion on the size of the device, please refer to supplementary note 3.

^b^Due to low latency (~ 18.3 ps) and fast response of our design^34^, state-of-the-art high-speed photodetectors with up to 50 GHz photodetection rate are applicable to our architecture. By assuming 20 GHz photodetection rate, the bit rate of our design, will be 20Gbps. For a brief discussion on the possible photodetection schemes, please refer to supplementary note 4.

Other than the advantages offered by the multi-layered metasurface-based architecture, the presented architecture benefits from its CMOS foundary-compatible silicon photonics platform, fabrication compatible with very matured silicon technology, simple configuration and compactness, and operation wavelength of 1550 nm. These make our design suitable for monolithic integration with other silicon-based photonic and electronic integrated circuits.

Also, the low-loss nature of this configuration results in low power consumption (for more details on the loss of system please refer to supplementary note 5) and its low-latency leads to high computational speed (see Table 3). Furthermore, the broadband Boolean logic operability of our design (over 60 nm in free-space wavelength) have been demonstrated and its capability for wavelength multiplexed parallel computation, which realize the full potential of optical computation, is studied. Although in our work, the diffractive network was trained for 60 nm bandwidth operation, higher-bandwidth logic gates are easy to be trained.

The main weakness of the presented design is its poor binary contrast. But it should be considered that for the purpose of full-wave electromagnetic verifications, it was tried to design a structure as compact as possible. Our design is composed of 500 neurons which is one fifth of the number of neurons in the proposal of^20^ that has 2520 neurons for multifunctional logic gate that functions as AND, NOT and OR. Therefore, it is expected that the binary contrast highly improves by using larger number of neurons.

Finally, the comparison of our logic gate with some recently reported researches in terms of architecture, dimensions, operation wavelength, operation bandwidth, contrast ratio, etc. is summarized is Table 4. The main advantage of our design over other works^1–15,17–19,35–37^ is that a single structure with the same structural and geometrical parameters can be used for all logic operations. Furthermore, comparing to the logic gate proposals named in Table 4, the other benefits offered by our logic gate are its on-chip design, operation wavelength of 1.55 µm, capability for broadband operation which is suitable for wavelength division multiplexing, and high computational speed (up to 50 Gbps).

**Table 4:** Comparison between our design and some of the state-of-the-art optical logic gates.

Ref. no.	Device architecture	Gate	Operation wavelength	Dimensions	Bit rate	Contrast ratio (dB)	Operation bandwidth
^12^	Si wire $\Psi$ gate	AND, NOR, XNOR	1.55 µm	3 µm × 3 µm	20Gbps	> 9	30 nm
^19^	L-shaped Optical slot nano-antenna	(AND, OR) or (NOT, NOR, NAND) depending on the orientation of the L-shaped antenna	800 nm	300 nm × 300 nm	–	> 13.27	230 nm
^13^	Mach–Zehnder interferometer switch	All seven logic gates but using different cascading schemes	1.55 µm	50 µm × 21 µm	25Gbps	–	–
^2^	Coupled nonlinear photonic crystal waveguides	AND, NOT	–	–	–	20	–
^14^	Two-dimensional photonic crystal	All seven logic gates but using different number of basic gates	2.5793 µm (116.31THz)	< 150 µm × 45 µm	–	> 9.54	–
^3^	Micro-resonator	AND	1.55 µm	1.5 mm × 1.5 mm	5kbps	11	–
^11^	Crossbar InP and AlGaAs nanowire networks	AND, OR, NAND, NOR	532 nm and 730 nm	Subwavelength scale	–	–	–
^16^	Curved silver grating	All seven logic gates	633 nm	≈ 10 µm × 10 µm	–	< 29	100 nm
This work	On-chip diffractive optical neural network	AND, OR, NOT (with the capability to be trained for all seven logic operations)	1.55 µm	100 µm × 1850 µm	20Gbps	< 10.82 (based on 2.5D FDTD)	60 nm (capable to be trained for larger bandwidths)

## Conclusion

In conclusion, a simple, integrable, low-loss, high-speed and broadband on-chip multi-functional optical logic gate capable of performing AND, NOT, and OR functions have been demonstrated using a diffractive optical neural network which is composed of cascaded on-chip 1D-metasurfaces. Due to broadband operation of the proposed logic gate, it is capable for wavelength multiplexed parallel computation, which helps to realize the full potential of optical computing. While the proposed logic gate shows poor binary contrast, a larger network composed of higher number of neurons can be trained to surmount this problem.

## Supplementary Information

Supplementary Information.

## Data Availability

All data generated or analyzed during this study are included in this published article (and its Supplementary Information files).
