# Supplementary material for: Realization of optical logic gates using on-chip diffractive optical neural networks
Source: Sci Rep. 2022 Sep 21;12:15747. doi: 10.1038/s41598-022-19973-0 (PMC9492711; doi:10.1038/s41598-022-19973-0)
Supplement: Supplementary file 1 — Supplementary Information. [file 41598_2022_19973_MOESM1_ESM.docx]

Supplementary information for

Realization of optical logic gates using on-chip diffractive optical neural networks

**Sanaz Zarei and Amin Khavasi**

Department of Electrical Engineering, Sharif University of Technology, Tehran, Iran

[*szarei@sharif.edu](mailto:*szarei@sharif.edu)

This PDF file includes:

- Supplementary Note 1: Back-propagating the errors in the network
- Supplementary Note 2: Design verification
- Supplementary Note 3: Size of the metasystem
- Supplementary Note 4: Photodetection schemes
- Supplementary Note 5: Loss of the metasystem
- References

**Supplementary Note 1:** Back-propagating the errors in the network

To compute the gradient of cost function with respect to all learnable parameters, the chain rule is used:

(S1)

where $\vec{\phi}^{m,\lambda_{j}}$ and $\vec{T}^{m,\lambda_{j}}$ denotes an N×1-dimensional vectors containing the diagonal entries of the matrices $\phi^{m,\lambda_{j}}$ and $T^{m,\lambda_{j}}$, respectively and symbol denotes element-wise multiplications. and can be found easily from Fig. 2 of the main article. To compute and , first the derivatives and (derivatives at a single spatial location *s'* on the *m'*th metaline) are calculated using the chain rule again:

(S2)

where Writinger Calculus for computing derivatives with respect to a complex variable is employed. denotes the real part of the expression enclosed in brackets, and $^{+}$ indicates the adjoint of a matrix. The derivatives and can be calculated using equations (1) and (3) in the main article, as follows:

(S3)

in which:

(S4)

If we assume that $a^{\lambda_{j}}=E^{out,\lambda_{j},f_{k}}\otimes(I^{\lambda_{j},f_{k}}-I^{des,\lambda_{j},f_{k}})$ is the adjoint field, then by substituting equations (S3) in equations (S2) and taking the adjoint of the resulted equations, the following equations are achieved:

(S5)

Once $\frac{dC}{dw^{m}}$ is computed, the learnable parameters can be updated at each iteration as follows:

$w^{m}=w^{m}-\alpha\frac{dC}{dw^{m}} \forall m$ (S6)

where $\alpha$is the learning rate.

**Supplementary Note 2: Design verification**

As was mentioned in the main text of the article, the challenge is designing an optical logic gate with 100% consistency between its numerical performance and the performance it shows when simulated with a commercial full-wave electromagnetic software. To achieve this goal, we numerically trained a number of diffractive networks and verified their performance with Lumerical 2.5D FDTD. Table S1 reports the results of these investigations. For the first demonstration of optical logic gates AND, NOT and OR, a DONN with three diffractive layers (metalines) is designed. Each metaline contains 200 meta-atoms (neurons). The length of each metaline is 100µm, and the distance between two successive layers is 200µm, and after light exits the third metaline, it propagates 200µm until it reaches the output line of the network with two linearly-arranged detection regions. The length of each detection region is 4µm and the distance between the two regions is 12µm. Like before, it is assumed that the distance between the input layer and the first metaline is zero. The Training process is similar to the one described in the “Results” section of the article. This optimization problem involves 600 design variables (200 variables per metaline).

Once the diffractive network is trained, in order to verify the performance of the obtained optical logic gates, the designed metasystem is implemented in Lumerical Mode Solution and simulated using its 2.5D variational FDTD solver. Table S1 describes the characteristics of the designed metasystem as well as its numerical performance and its verification using Lumerical Mode Solution 2.5D FDTD (design 1). The simulations indicate 90% match between numerical testing results and full-wave simulation results. In design 2, each neuron value is approximated by a slot group composed of two identical slots, in order to reduce the influence of mutual interference between adjacent slots. All the design metrics for this design are similar to design 1, except otherwise the number of neurons in each metaline which is equal to 100 and the number of slots in each neuron (that is equal to two identical slots). For this design, the consistency between the two obtained results reduces to 70%. This can be due to decreased number of neurons in this metasystem (300 neurons instead of 600 neurons for previous design). For design 3, again each metaline contains 200 meta-atoms and according to Fig. 3 of the article, the distance between the diffractive layers as well as the distance between the last diffractive layer and the output layer is assumed to be 300µm. As is seen in Table S1, also for this design, there is 90% match between numerical testing and full-wave simulations. If we further approximate each neuron value by a slot group composed of two identical slots, in order to reduce the influence of mutual interference between adjacent slots, design 4 will be obtained. All the design metrics for design 4, except otherwise the number of neurons in each metaline which is equal to 100 and the number of slots in each neuron (that is equal to two identical slots), are similar to design 3. Again, for this design, the consistency between the two obtained results reduces to 80% because of the decreased number of neurons in this metasystem with respect to design 3, which indicates that the task cannot be successfully accomplished using only three learnable diffractive layers.

Therefore, we increase the number of layers to five diffractive layers (design 5), again with 200 meta-atoms in each layer. For the first demonstration of a five-layered DONN and for the sake of compactness, the distance between two successive layers and the distance between the last layer and the output layer is set to be 50µm. The other design parameters for this metasystem are like design 1. Because in the method described in subsection “Modeling” of the article, the distance between adjacent layers should be large enough so that only the far-field from one metaline layer reaches the next layer, this method cannot be applied for designing this metasystem. Because in this metasystem, the distance between the layers is only 50µm which is even less than the metaline length (100µm). Therefore, for training this DONN, the computational method presented in [24-25] is utilized. For this design, the simulations predicate 80% matching between numerical testing results and full-wave simulation results. For design 6, all the design parameters are chosen similar to design 1. For this design, verifications with Lumerical Mode Solution 2.5D FDTD provide 90% fitting to the numerically trained diffractive model. For design 7, we keep all the design parameters like design 6 except that in this design we approximate each neuron value by a slot group of two identical slots (which means each diffractive layer contains 100 meta-atoms). For this design, the matching between numerical testing results and full-wave simulations reduces to 70%, which again can be due to reduced number of meta-atoms in this metasystem with respect to design 6 (500 meta-atoms versus 1000 meta-atoms). In design 8, the distance between the layers and the distance between the last layer and the output layer is changed to 300µm, but all other design metrics are kept similar to design 6. For this design, complete matching between numerically trained model and Lumerical Mode Solution 2.5D FDTD is achieved (see Table S1). If we move a step forward in design 9 and approximate each neuron value by a slot group of two identical slots and keep all other parameters like design 8, again complete matching between numerically trained model and Lumerical 2.5D FDTD is achievable (design 9 is the design which is studied in Fig. 5 of the manuscript). Designs 10 and 11 in Table S1 are alike design 1 except otherwise that the distance between the two detectors are 32µm and 22µm, respectively. Similarly in design 12, only the distance between the detectors in design 5 is changed to 32µm. Also, all design parameters in designs 13 and 14 are similar to designs 6 and 7, respectively, excluding the distance between the two detection regions that is equal to 22µm in these two designs. The matching percentage between numerical simulation results and full-wave simulation results for these designs are also brought in Table S1.

| **Table S1. The characteristics of a number of diffractive neural networks that operate as a multi-functional logic gate and the comparison between their numerical testing results and full-wave simulation results.** | | | | | | | | | |
| --- | --- | --- | --- | --- | --- | --- | --- | --- | --- |
| Design | Number of layers | Number of neurons in each layer | Number of slots in each neuron | Length of the layers | Distance between layers | Distance between the last layer and output layer | Length of detection regions | Center to center distance between detection regions | Percentage of matching between numerical predictions and full-wave simulations |
| **1** | 3 | 200 | 1 | 100µm | 200µm | 200µm | 4µm | 12µm | 90% |
| 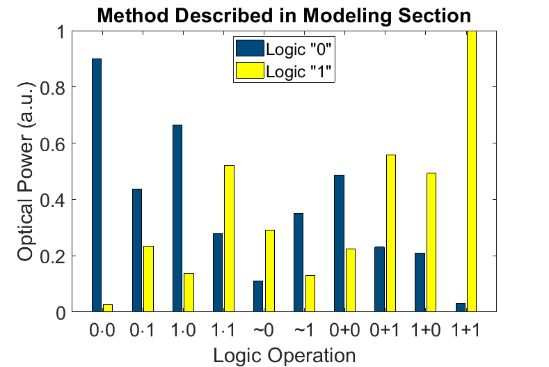 | | | | | 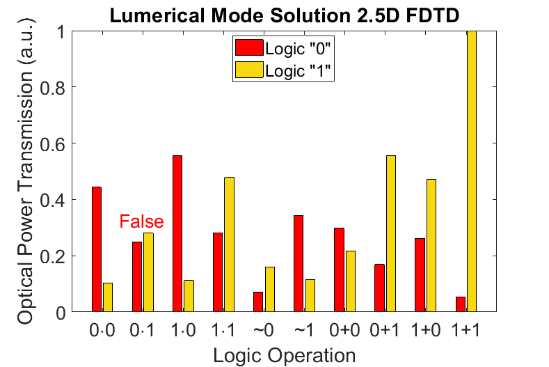 | | | | |
| **2** | 3 | 100 | 2 | 100µm | 200µm | 200µm | 4µm | 12µm | 70% |
| 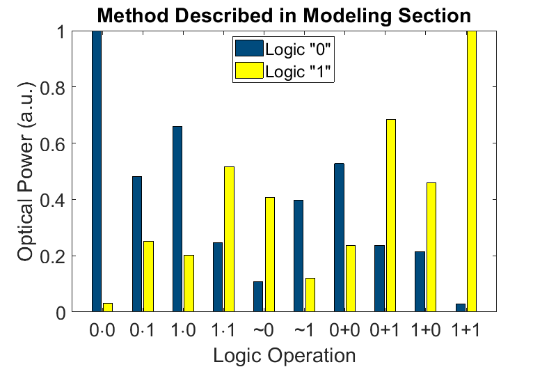 | | | | | 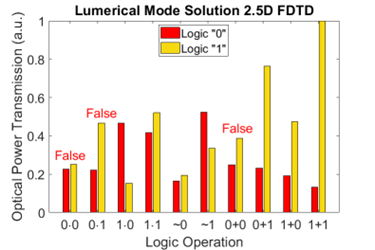 | | | | |
| **3** | 3 | 200 | 1 | 100µm | 300µm | 300µm | 4µm | 12µm | 90% |
| 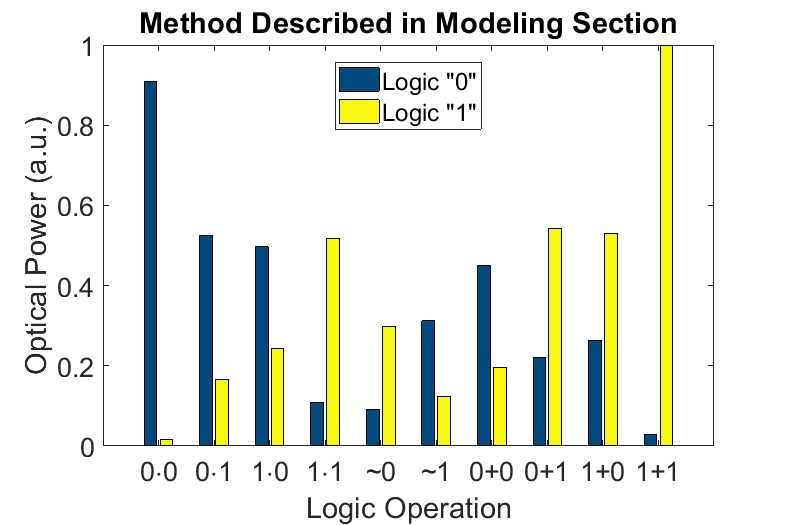 | | | | | 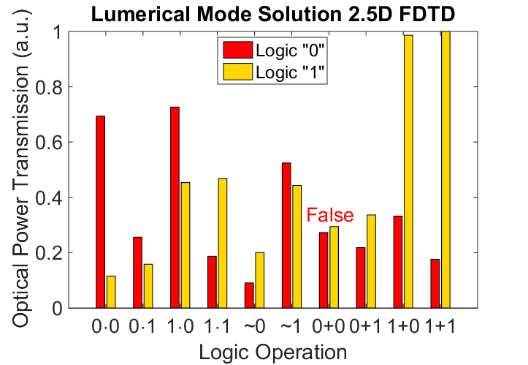 | | | | |
| **4** | 3 | 100 | 2 | 100µm | 300µm | 300µm | 4µm | 12µm | 80% |
| 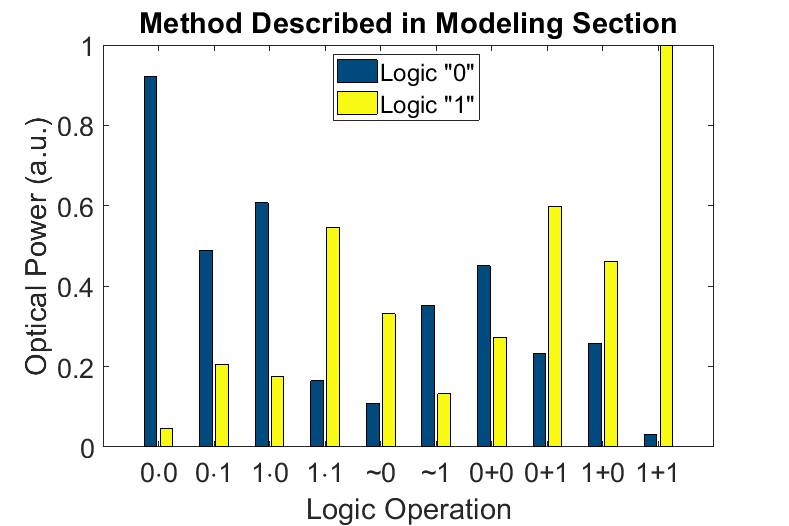 | | | | | 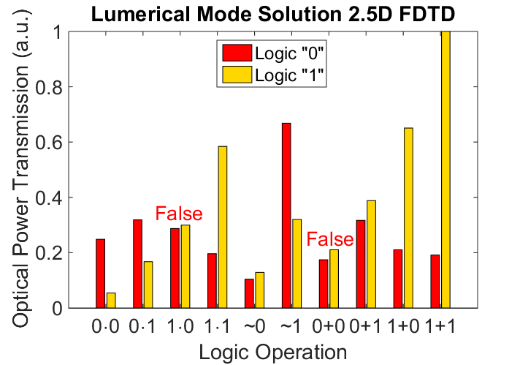 | | | | |
| **5** | 5 | 200 | 1 | 100µm | 50µm | 50µm | 4µm | 12µm | 80% |
| 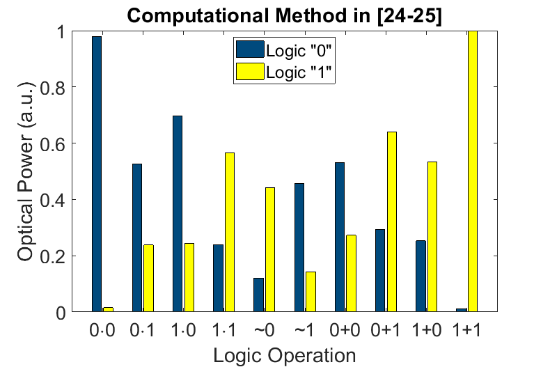 | | | | | 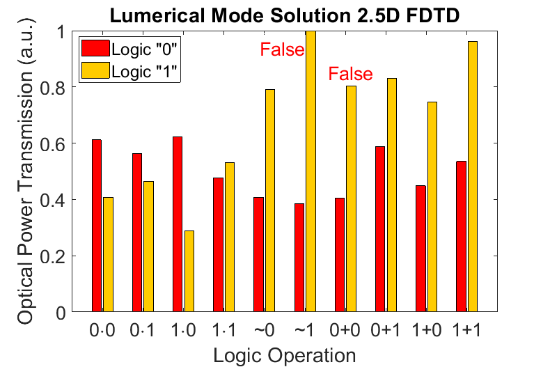 | | | | |
| **6** | 5 | 200 | 1 | 100µm | 200µm | 200µm | 4µm | 12µm | 90% |
| 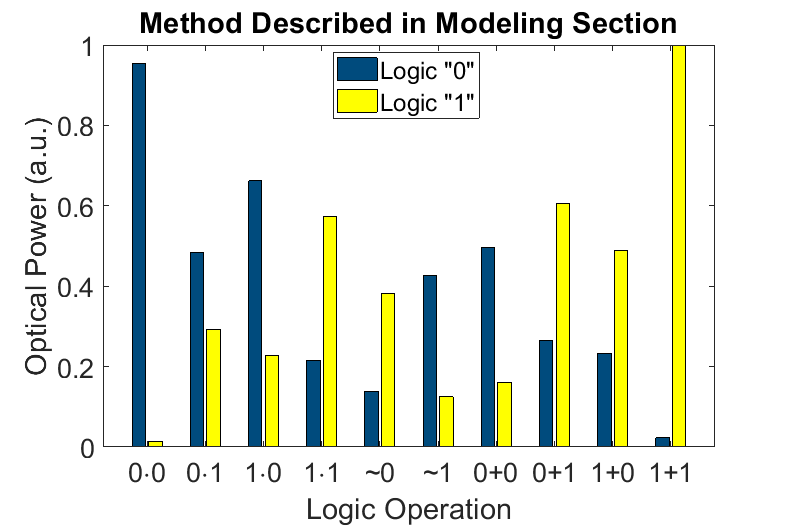 | | | | | 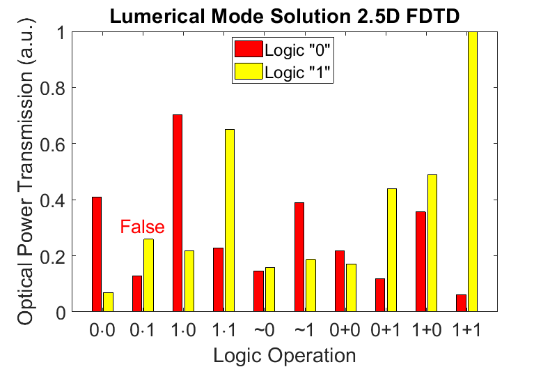 | | | | |
| **7** | 5 | 100 | 2 | 100µm | 200µm | 200µm | 4µm | 12µm | 70% |
| 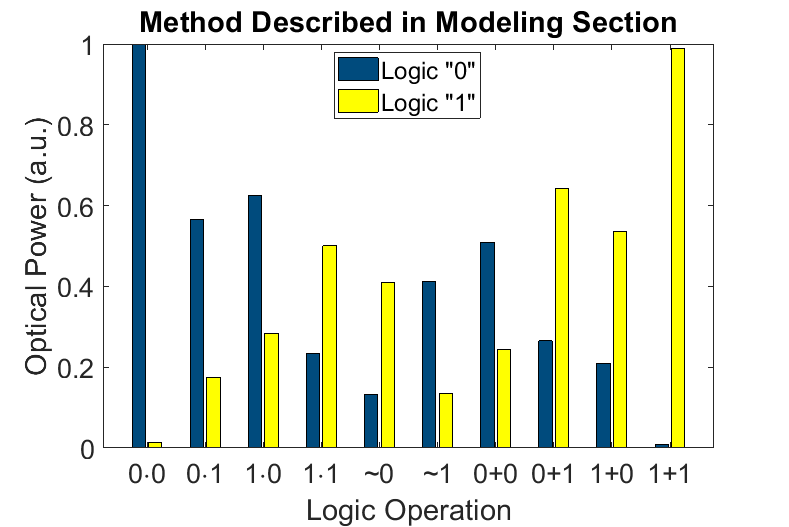 | | | | | 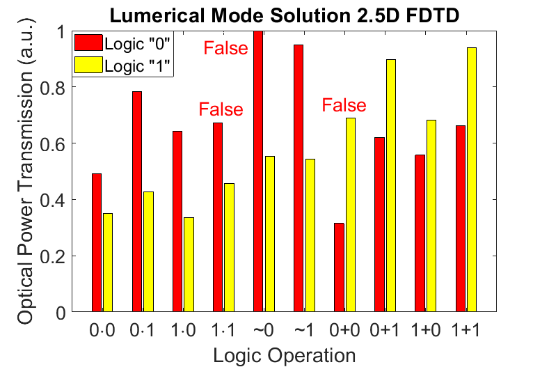 | | | | |
| **8** | 5 | 200 | 1 | 100µm | 300µm | 300µm | 4µm | 12µm | 100% |
| 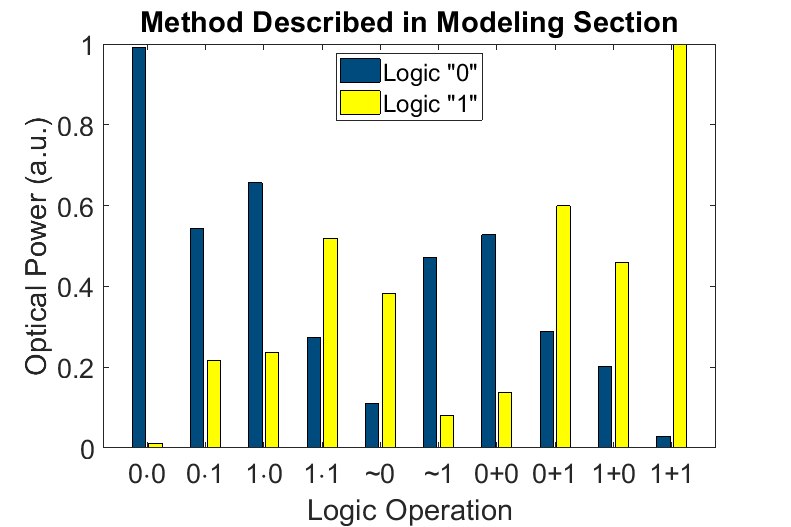 | | | | | 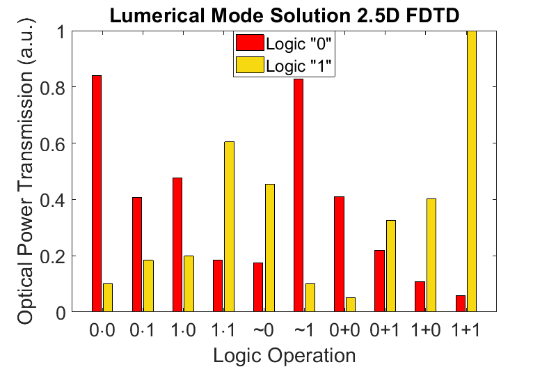 | | | | |
| **9** | 5 | 100 | 2 | 100µm | 300µm | 300µm | 4µm | 12µm | 100% |
| 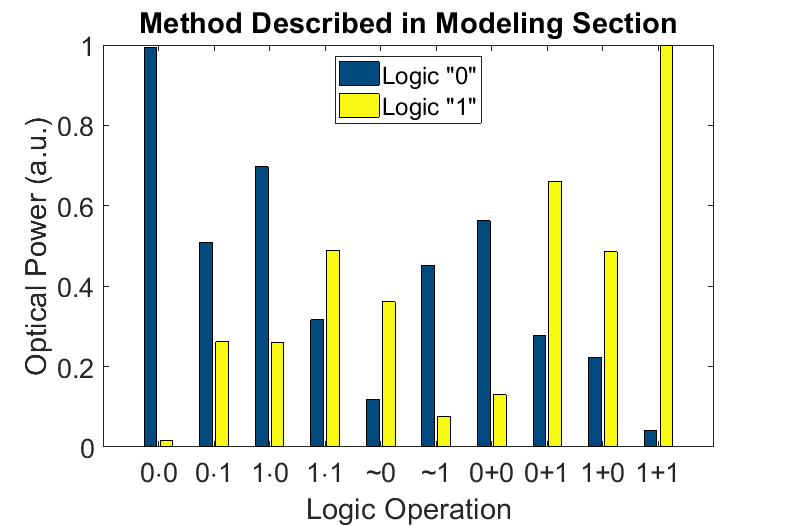 | | | | | 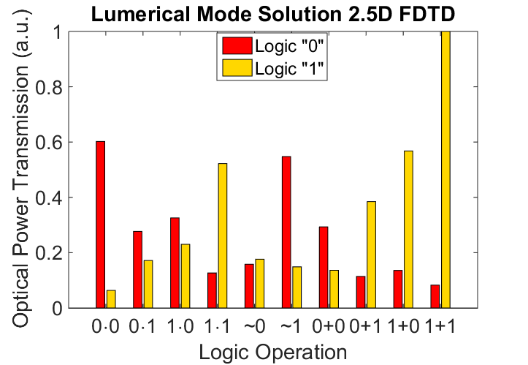 | | | | |
| **10** | 3 | 200 | 1 | 100µm | 200µm | 200µm | 4µm | 32µm | 80% |
| 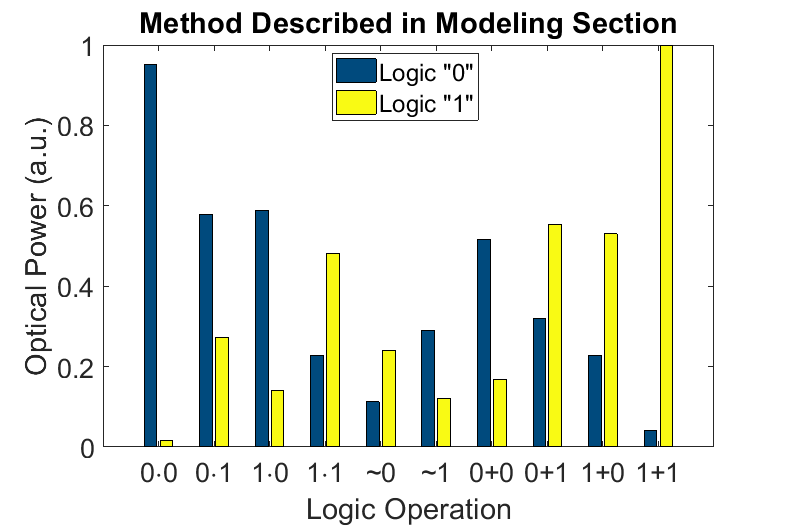 | | | | | 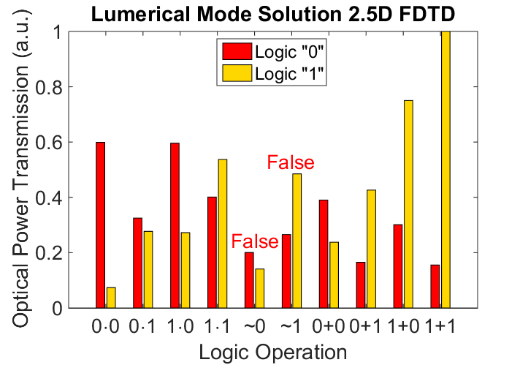 | | | | |
| **11** | 3 | 200 | 1 | 100µm | 200µm | 200µm | 4µm | 22µm | 90% |
| 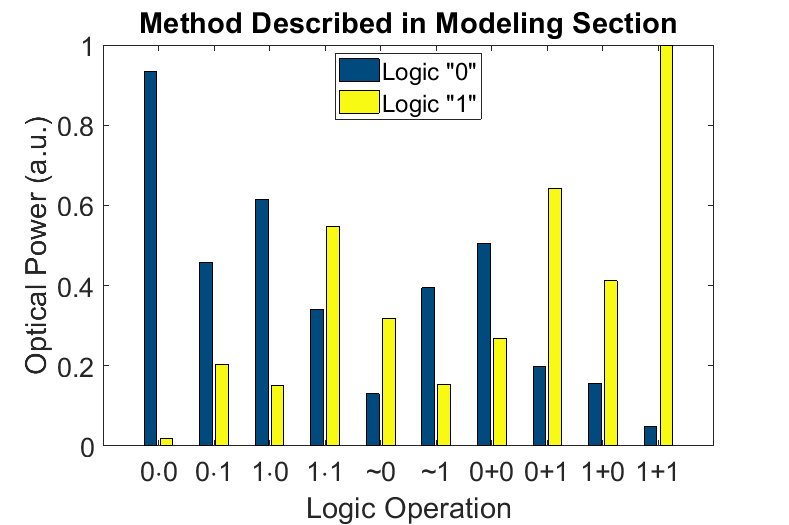 | | | | | 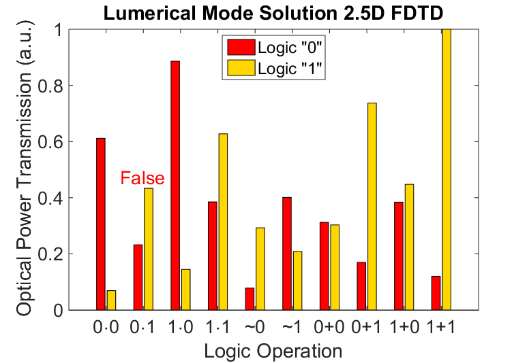 | | | | |
| **12** | 5 | 200 | 1 | 100µm | 50µm | 50µm | 4µm | 32µm | 60% |
| 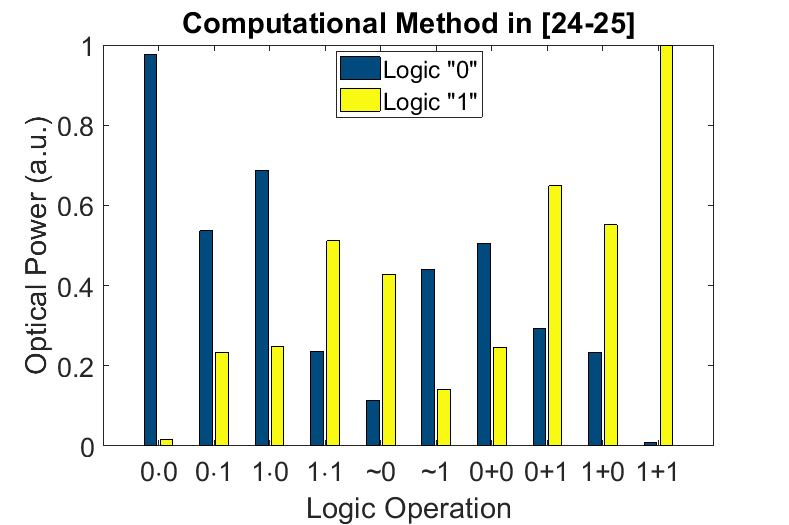 | | | | | 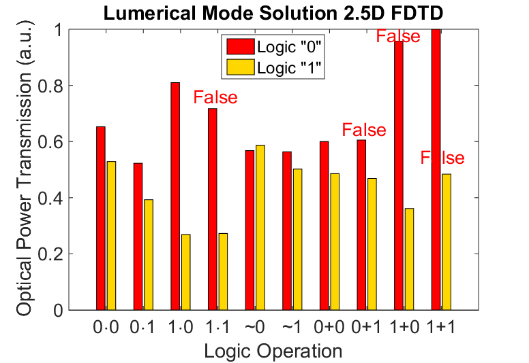 | | | | |
| **13** | 5 | 200 | 1 | 100µm | 200µm | 200µm | 4µm | 22µm | 90% |
| 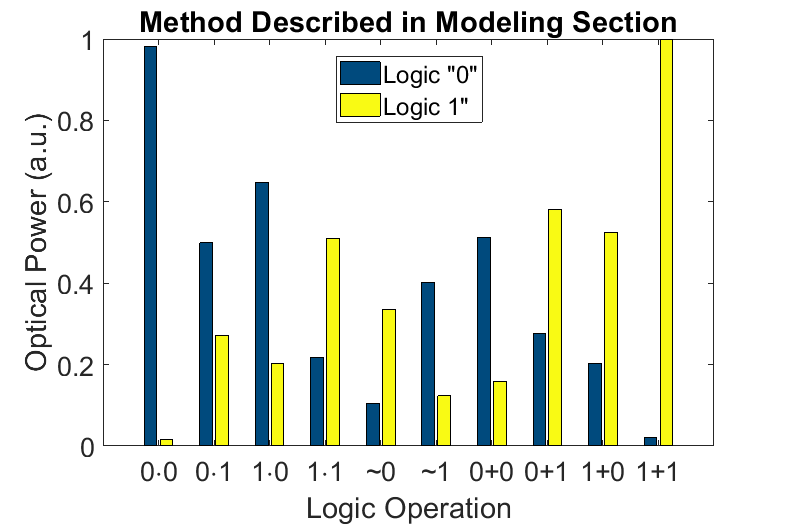 | | | | | 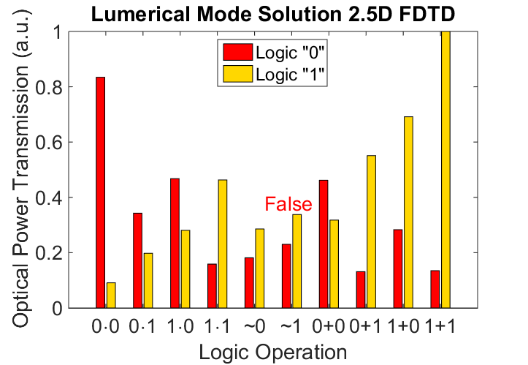 | | | | |
| **14** | 5 | 100 | 2 | 100µm | 200µm | 200µm | 4µm | 22µm | 80% |
| 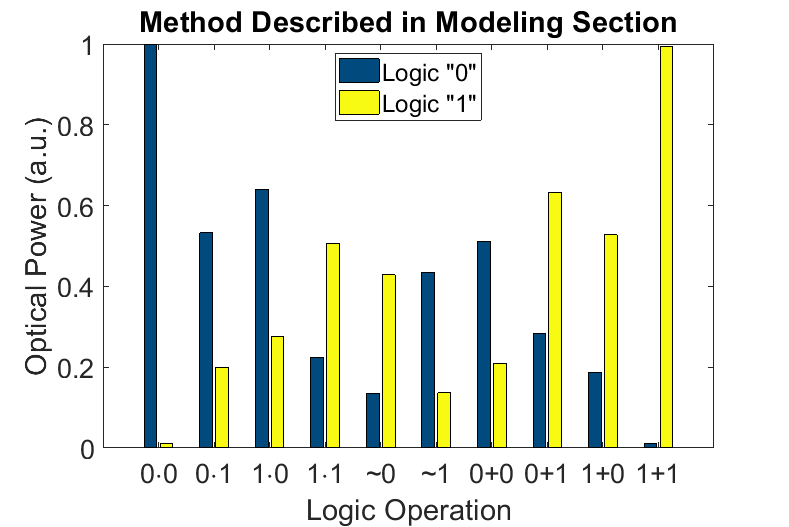 | | | | | 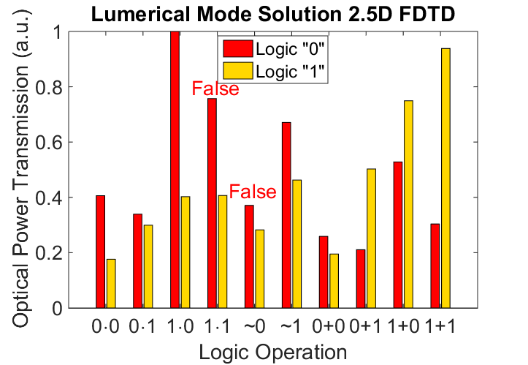 | | | | |

**Supplementary Note 3: Size of the metasystem**

As is seen in Fig. S1, the distance between the input excitations and the output detections is 1850µm, which is the sum of the length of input waveguides (20µm) and tapers (280µm), the distance between two successive metalines (300µm) multiplied by 4 (number of metalines minus 1), the distance between the fifth metaline and output line (300µm), and the thickness of the metalines (10µm) multiplied by five (number of metalines).

| 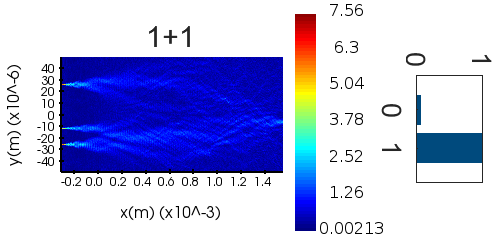 |
| --- |
| (a) |
| 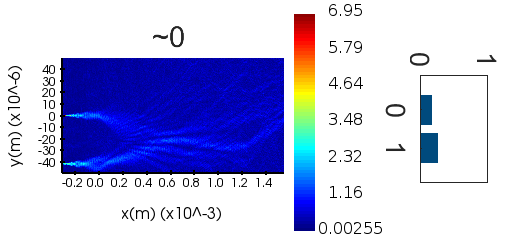 |
| (b) |
| Fig. S1. The x-y view of the electric field profile in the whole multifunctional logic gate structure for (a) logic operation 1+1 (1 OR 1) which has the highest contrast ratio at the output (Fig. 5(c)), (b) logic operation ~0 (NOT 0) which has the lowest contrast ratio at the output (Fig. 5(c)). The length of input waveguides and tapers are 20µm and 280µm, respectively. The length of each metaline is 100µm, the distance between two successive layers is 300µm, and after light exits the fifth metaline, it propagates 300µm until it reaches the output line. |

As is known, the number of metalines (hidden layers), the number of meta-atoms (neurons) in each metaline, the distance between two successive metalines and the distance between the last metaline and the output layer are the design parameters, which will affect the distance between the input excitations and the output detections and the final size of the device.

As is stated in subsection “Design principle” of the manuscript, in the training process and for simplicity, it is assumed that when an input is applied to one of the waveguides, its value at the exit of the taper corresponding to that waveguide (or equivalently at the input layer) is "1". So, for designing the input waveguides and tapers for the DONN studied in Fig. 5 of the manuscript, it is tried to fulfill this assumption at the exit of the tapers as much as possible. However, in experiments, whatever the fields at the exit of the tapers are, the network can be trained for those field profiles.

The parameters shown in Table S2 are used in the simulations that end-up to the results shown in Fig. S1. The simulations are performed only for the best (1 OR 1) and worst (NOT 0) logic operations in terms of contrast ratio between the logical outputs “0” and “1”. For both cases, Lumerical Mode Solution 2.5D FDTD simulations indicate that the network functions accurately.

| **Table S2. Input waveguides and tapers characteristics used in the simulations shown in Fig. S1.** | | |
| --- | --- | --- |
|  | **width** | **length** |
| Input A and B waveguides | 1µm | 20µm |
| Input A and B tapers | 1µm (input), 16µm (output) | 280µm |
| Control Inputs waveguides | 0.75µm | 20µm |
| Control Inputs tapers | 0.75µm (input), 12µm (output) | 280µm |

A related point to consider is that for the fabrication of the designed logic gate, all the designed patterns (e.g., metalines, waveguides and tapers) can be defined using electron beam lithography, followed by a single step dry etch procedure.

**Supplementary Note 4: Photodetection schemes**

In [S1] a layout based on waveguides is suggested for output detection. In this layout, a single-mode fiber probe collects optical outputs and sends them to an InGaAs photodiode and optical power meter (Newport 818-IG-L-FC/DB and 1830-RGPIB). An alternative layout based on taper-guides is suggested for output detection in [S2]. In this detection scheme, the outputs are monitored using two dual-channel optical power meters with minimum power detection limit of -75dB. These detection schemes are also applicable to our work.

Furthermore, some on-chip detection schemes [S3-S5] are utilized in the integrated optical neural networks based on Mach-Zehnder interferometers [S6-S8] mainly due to their benefits of high detection rate [S3] and small footprint [S4]. These detection schemes may also be applicable to our work. Especially, the Ge photodetector presented in [S3], which have advantages like zero-bias operation, high responsivity, and high detection speed, is suitable for the presented layout.

**Supplementary Note 5: Loss of the metasystem**

In Table S3, the loss imposed by each part of the system which is calculated by Lumerical software package is given:

| **Table S3. Power loss of each part of the system** | |
| --- | --- |
| **Component** | **Loss (dB)** |
| A logic input waveguide and taper | 0.36 |
| A control input waveguide and taper | 0.56 |
| Meta-atom | Maximum 0.35 |
| Total metasystem loss for calculating 1+1 (Fig. S1(a)) | 1.95 |
| Total metasystem loss for calculating ~0 (Fig. S1(b)) | 2.26 |

These values are calculated for the metasystems shown in Fig. S1. The loss of waveguides and tapers are calculated (by Lumerical Mode Solution 2.5D FDTD) based on the design parameters presented in Table S2 at the wavelength of 1.55μm. The maximum power transmission loss computed for each meta-atom (by Lumerical FDTD) is approximately 0.35dB at the wavelength of 1.55μm. Although due to the parallel interaction between electric field and meta-atoms on a metaline layer, the maximum power transmission loss of each layer can be regarded as 0.35dB, in practice, the maximum power loss of metalines may be higher than the above-mentioned value. The reason is that the local periodic approximation is not absolutely true over all regions, which means that the output electric field transmission phase and amplitude associated with a meta-atom not only depends on its own geometrical parameters, but also on the geometrical parameters of its neighbors.

**References**

[S1] Wang, Z., Chang, L., Wang, F., Li, T., Gu, T. Integrated photonic metasystem for image classifications at telecommunication wavelength. *Nat. Commun.* **13**, 2131 (2022).

[S2] Fu, T., Zang, Y., Huang, Y., Du, Z., Huang, H., Hu, C., et al. Photonic machine learning with on-chip diffractive optics. 13 May 2022, PREPRINT (Version 1) available at Research Square [https://doi.org/10.21203/rs.3.rs-1550655/v1].

[S3] Vivien, L., Polzer, A., Marris-Morini, D., Osmond, J., Hartmann, J. M., Crozat, P., et al. Zero-bias 40 Gbit/s germanium waveguide photodetector on silicon. *Opt. Express* **20**, 1096 (2012).

[S4] Nozaki, K., Matsuo, S., Shinya, A., Notomi, M. Amplifier-Free Bias-Free Receiver Based on Low Capacitance Nanophotodetector. *IEEE J. Sel. Top. Quantum Electron.* **24**, 1 (2018).

[S5] Morichetti, F., Grillanda, S., Carminati, M., Ferrari, G., Sampietro, M. Non-invasive onchip light observation by contactless waveguide conductivity monitoring. *IEEE J. Sel. Top. Quantum Electron.* **20**, 292–301, (2014).

[S6] Shen, Y., Harris, N., Skirlo, S., Prabhu, M., Baehr-Jones, T., Hochberg, M., et al. Deep learning with coherent nanophotonic circuits. *Nat. Photonics* **11**, 441 (2017).

[S7] Hughes, T. W., Minkov, M., Shi, Y., Fan, S. Training of photonic neural networks through in situ backpropagation and gradient measurement. *Optica* **5**, 864 (2018).

[S8] Williamson, I. A. D., Hughes, T. W., Minkov, M., Bartlett, B., Pai, S., Fan, S. Reprogrammable electro-optic nonlinear activation functions for optical neural networks. *IEEE J. Sel. Top. Quantum Electron*. **26**(1), 1 (2020).
